# Plant and Salamander Inspired Network Attack Detection and Data Recovery Model

**DOI:** 10.3390/s23125562

**Published:** 2023-06-14

**Authors:** Rupam Kumar Sharma, Biju Issac, Qin Xin, Thippa Reddy Gadekallu, Keshab Nath

**Affiliations:** 1 Department of Computer Science and Engineering, Rajiv Gandhi University, Itanagar 791112, India; 2 Department of Computer and Information Sciences, Northumbria University, Newcastle upon Tyne NE1 8ST, UK; 3 Faculty of Science and Technology, University of the Faroe Islands, Vestara Bryggja 15, FO-100 Tórshavn, Faroe Islands; 4 School of Information Technology and Engineering, Vellore Institute of Technology & Engineering, Vellore 632014, India; thippareddy.g@vit.ac.in; 5 Department of Electrical and Computer Engineering, Lebanese American University, Byblos P.O. Box 36, Lebanon; 6 Zhongda Group, Haiyan County, Jiaxing 314312, China; 7 College of Information Science and Engineering, Jiaxing University, Jiaxing 314001, China; 8 Division of Research and Development, Lovely Professional University, Phagwara 144401, India; 9 Department of Computer Science and Engineering, Indian Institute of Information Technology, Kottayam 686635, India

**Keywords:** intrusion detection, network security, bio-inspired algorithm, evolutionary computing, ransomware

## Abstract

The number of users of the Internet has been continuously rising, with an estimated 5.1 billion users in 2023, which comprises around 64.7% of the total world population. This indicates the rise of more connected devices to the network. On average, 30,000 websites are hacked daily, and nearly 64% of companies worldwide experience at least one type of cyberattack. As per IDC’s 2022 Ransomware study, two-thirds of global organizations were hit by a ransomware attack that year. This creates the desire for a more robust and evolutionary attack detection and recovery model. One aspect of the study is the bio-inspiration models. This is because of the natural ability of living organisms to withstand various odd circumstances and overcome them with an optimization strategy. In contrast to the limitations of machine learning models with the need for quality datasets and computational availability, bio-inspired models can perform in low computational environments, and their performances are designed to evolve naturally with time. This study concentrates on exploring the evolutionary defence mechanism in plants and understanding how plants react to any known external attacks and how the response mechanism changes to unknown attacks. This study also explores how regenerative models, such as salamander limb regeneration, could build a network recovery system where services could be automatically activated after a network attack, and data could be recovered automatically by the network after a ransomware-like attack. The performance of the proposed model is compared to open-source IDS Snort and data recovery systems such as Burp and Casandra.

## 1. Introduction

The present-day security and defence mechanisms are a game between an attacker and defender of a network. This has challenged both the attackers and the network security experts to explore new robust techniques that could be dynamic and evolutionary in nature. This has led researchers to use different soft computing and optimization techniques over time with the Intrusion Detection System (IDS) installed in a network [1,2,3,4,5,6]. However, detection techniques have never been a foolproof approach to withstand new attacks as they have successfully infiltrated networks [7,8]. A comprehensive survey of how present-day crypto ransomware evades the detection techniques is available in the work of Alqahtani et al. [9]. In the work of Urooj et al. [10], the role of Deep Learning and Machine Learning is discussed both in the performance of the attack and detection of the attack. Henry et al. [11] have demonstrated that using a bidirectional LSTM network, a potential network anomaly could be detected in a short session of time. However, one of the fundamental challenges in the applicability of Machine Learning- (ML) and Deep Learning- (DL) based intrusion detection systems is that most of them are trained using an almost decade-old NSL-KDD dataset. Attacks have advanced recently, but few updated datasets are found that are recent and public. Ferriyan et al. [12] in their work have developed a public dataset, HIKARI-2021, that could immensely help the research communities in upgrading the learning attributes for attack detection. It can be concluded more generically that with all the known computing techniques of intrusion detection, it is not possible to guarantee an attack-free network. Therefore, it is equally important to design an intrusion response system hand in hand with an intrusion detection system [13,14,15,16,17,18]. Different intrusion response systems such as ACBIRS [19] and PRE [20] are found in the literature with the common observation that the response engine proposed or implemented is after the alert generated by the detection engine. However, all the approaches seen in the literature overlook one fundamental fact. If the detection fails in the first place, then the response engine may never be triggered. It is therefore important to look into natural evolutionary organisms to understand how these organisms withstand defence against these attacks so precisely and in constrained time. The primary concentration of studies of bio-inspired algorithms is restricted to optimization problems. Algorithms such as GA, ACO, PSO, AIS, etc., used in IDS are used to contribute to the enhancement of feature extraction for better classification results. However, no results were found that concentrated on the design possibility of an intrusion detection system itself that could be an analogy of the evolutionary defence mechanisms of the organisms. The contribution of this research is to bridge this gap and explore the different defence mechanisms of organisms, and finally pick one to implement and test on a network model. Biological organisms have an inherent capability to evolve, self-organize, and self-heal without any centralized control. They also inherently respond to changing environmental conditions by exhibiting collaborating, self-organizing and adaptation properties. This paper tries to address the specific issue of data and services recovery after failed detection of a network attack. The three-layer defence model of plants is taken as an inspiration for network attack detection along with the limb regeneration process in salamanders for data recovery towards ransomware-like attacks in the network. As with multilevel defence in plants, a multi-layer defence is proposed and implemented to ensure that attacks, both known and unknown, can be detected in the network. The positional identity storage mechanism used by the salamander for limb regeneration is used for the implementation of data recovery in a network. Due to their natural ability to evolve with cooperation and interaction with its surroundings, evolutionary algorithms have gained the interest of the research community in spite of the wide popularity and applications of Machine Learning (ML) and Deep Learning (DL) techniques in the field of IDS. A comparative outline between ML and Evolutionary Algorithms [2] is listed in Table 1.

The paper is further organized by: Section 2 conducting research into bio-inspired algorithms, followed by Section 3 describing plant defence mechanisms along with describing limb regeneration in salamanders. The paper proposes models based on plant defence and salamanders. It then incorporated fuzzy logic and detection complexity to experiment and analyze the model. Finally, it evaluates and provides a conclusion towards the method.

## 2. Related Work

A lot of research is being put into exploring nature and designing nature-inspired algorithms for optimization. Most of the acts in nature are observed to achieve marvellous things collectively rather than with limited individualistic capacity. The results of such studies have led to the proposal of Ant Colony Optimization (ACO), Genetic Algorithm (GA), Artificial Immune System (AIS), Swarm Intelligence (SI), Bat algorithm, Firefly algorithm, etc. In this section, such nature-inspired algorithms in the context of Intrusion Detection Systems are explored and listed.

### 2.1. Genetic Algorithm

The genetic algorithm optimization technique considers the natural evolution of genetics. A fit set of individuals are extracted by conducting crossover, mutation and selection processes on the given population, which are the set of optimal solutions. Genetic Algorithm (GA) and Genetic Programming (GP) are considered to be among the most widely used evolutionary algorithms. In these approaches, the objective of creating new offspring from the existing population that turns up to be more fit and representative of the solution is considered. Different nature-inspired operators, such as crossover, mutation, etc., are used to conclude an optimal solution. For evaluating the fitness of a solution candidate, a fitness function is defined. It is, however, found that GA is less efficient with high dimensional, multi-modal and complex problems where computing and evaluating the fitness function is expensive computationally and in time. Applications of GA can be found in numerous ML-based IDS. In the work of Aslahi-Shahri et al. [21], GA is used for SVM kernel parameter optimization used for attack classification in IDS (for rich widgets—https://github.com/rds1983/Myra (accessed on 22 October 2022)). By doing so, the authors have proved the improved accuracy. Most relevant features are extracted using GA, and then the features are grouped based on the priorities. The features with the lowest priorities are ignored as they do not carry much significance in the classification. The high-priority features group is fed to SVM for classification. Hashemi et al. [22] have used GA to extract the best decision rules of ID3 used together with Naive Bayes for classification. The fitness function defined for testing the quality of the rule is given by the following equation.

Fitness=a/A−b/B

where ‘*a*’ signifies the attacks that are correctly identified, ‘*A*’ signifies the total number of attacks in the dataset that are considered, ‘*b*’ signifies the total number of false positives and ‘*B*’ signifies the total number of normal instances in the dataset.

Chiba et al. [23] have proposed IGA (Improved Genetic Algorithm) to build a Deep Neural Network (DNN)-based IDS. The authors have used it to extract the relevant features to use in DNN. The IGA randomly generates a number of nodes of DNN’s hidden layer, values of the learning rate and the momentum. IGA is found to successfully discover appropriate values of the learning rate and the momentum. With 20k instances of both training and testing sets, the accuracy of the DNN using IGA is found to be 99.86% with a precision value of 99.93% and a detection rate of 99.80%. Similar to GA, Genetic Programming has been used, where software is programmed to behave and think with natural aspects. The authors in [24], have compared the performance of different classifiers along with and without feature selection (FS). FS enhanced the performances of the classifiers.

### 2.2. Swarm-Based Algorithms

**Figure 1:**
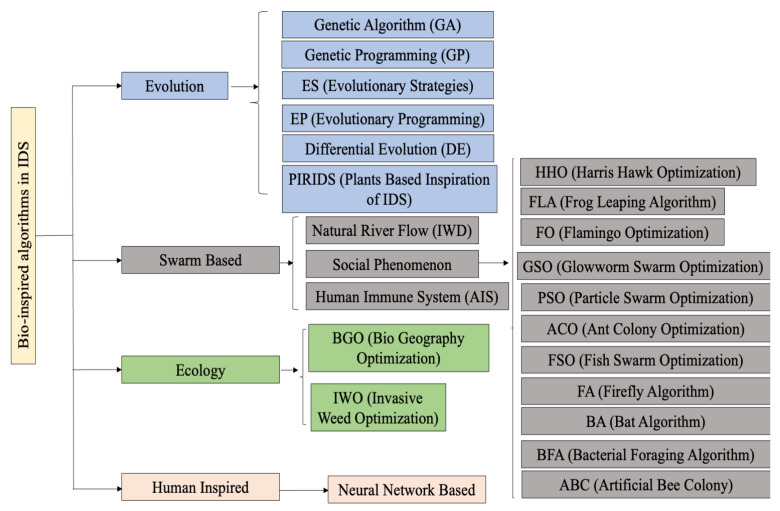
Taxonomy of Evolutionary Computation used in IDS.

The term ‘Swarm Intelligence’ became more mature after its first context use by Beni et al. [25] in their work on cellular robotics systems. It refers to the collective intelligence exhibited while interacting with nature by animals such as birds, fish and ants. Individualistic intelligence is limited but collectively can be a marvel in achieving certain goals. Swarm intelligence originated from the behaviour of living organisms such as ants, bees, birds, fish, etc. Swarm intelligence considers evolutionary properties and is built on properties such as adaptability and cooperation. The communication between the members of a swarm could be direct or indirect. A taxonomy of Evolutionary Computation used in IDS is shown in Figure 1.

To model individual behaviour in a swarm, certain principles can be inferred that are listed below [2,26,27].

- Proximity Principle: This indicates the necessity and importance of the swarm agents to respond to even small variances in the environment and pass the information to other agents such that an overall response can be generated to the variance of the environment.
- Quality Principle: Consequence to responding to the variance in the environment, the members of the swarm should also ensure the quality of food, safety in location, proper path route towards the food source, etc.
- Principle of Diverse Response: Whenever there is an environmental change, the resources should be available to each swarm agent, and therefore, the resources should be distributed in such a manner prior.
- Principle of Stability: Each swarm agent should show deterrence to changes in its behavioral characteristics even when environmental conditions are fluctuating.
- Principle of Adaptability: When the mode of environment changes, the members of the swarm should be able to adapt to the new changes.

#### 2.2.1. Ant Colony Optimization (ACO)

The authors Aghdam et al. [28] have proposed an ant colony-based approach for FS. An ant population was the feature vector generated from the training set. Once the population set was generated, the logical pheromones were generated. The pheromone indicates the strength of a feature being included in the feature vector set. If a feature proves to influence in attack detection, then the pheromone concentration of that feature increases along with the probability of the ant carrying that feature in the feature vector. The experiments were carried on KDD Cup 99 dataset, and the detection rate of Denial of Service (DoS) attack was 99.78%, with an overall accuracy of 98.9% and a false positive (FP) rate of 2.59%. An increase in ACO iterations increased the true positive (TP) rate and subsequently decreased the FP rate. The authors Botes et al. [29] have evaluated the performance of the ATM (Automated Tune Models) classifier with respect to a decision tree (ID) domain. The Java library available from the MYRA GitHub repository was used with the tuning of the following parameters.

- Colony size (CS): This indicates the population of the ants within an ant colony.
- Indicates the number of iterations that occur until an optimal global tree is discovered.
- Indicates the factor by which pheromone evaporates for each entry in the pheromone matrix.

**Figure 2:**
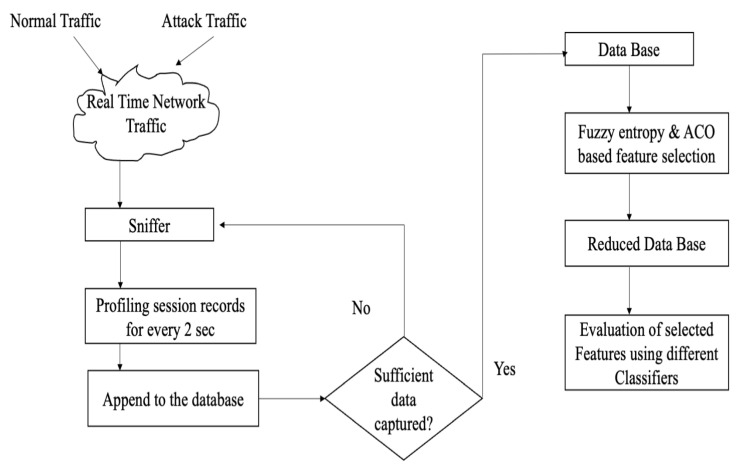
Real time feature extraction for IDS [31].

Using the ACO approach, optimal parameters for the classifier are identified. It was discovered that a colony population size of 5 and evaporation factor of 0.9 performed better than all other combinations. The classifier result was found to converge after 200 iterations. The important conclusion from the experiments was that the classifier cost was reduced with a reduction in the size of the colony with a limited or little compromise on accuracy. If the evaporating factor is reduced, then the classifier produces more accurate results. Reducing the number of maximum iterations produces less appropriate results. In the work of Varma et al. [30], the authors have demonstrated how a Fuzzy-entropy-based heuristic approach for ACO can be used to extract the smallest set of real-time traffic features for IDS. Fuzzy entropy is designed and based upon fuzzy equivalence relation. A fuzzy equivalence relation is different from rough set equivalence relation. The methodology of the proposed model is shown in Figure 2.

As can be seen in Figure 2, network traffic that is a mixture of normal and attacks is gathered. The collected network data are being appended to the database. It is then from this database that the minimal network feature set is extracted using Fuzzy entropy and ACO. Once the feature set is extracted, it is then evaluated using different classifiers. The following classifiers and their respective evaluation results are shown below.

- J48—99.45%;
- Random Tree—99.83%;
- Random Forest—99.34%;
- JRIP—99.58%.

It can be seen that the proposed work has proven convincing results, but Random Tree outperforms all others.

#### 2.2.2. Particle Swarm Optimization (PSO)

PSO is inspired by swarming behavior seen in fish and birds. The swarm is an accumulation of particles, and each particle is defined by its spatial feature in a multidimensional search space to represent a potential solution to a problem. The movement of a particle is constrained by its velocity and memory of locations where the objective of the search space is to explore the objective function with minimum cost. While applying PSO in IDS, it is important to consider different factors such as the search space, particle’s initialization pattern, swarm size, and particle dynamics, and they are established in the work of El Bekri et al. [32]. The authors have tried to combine data-mining techniques together with PSO for intrusion detection. In the proposed algorithm, each swarm particle is initialized with a certain position and velocity. Until the maximum iterations are received, the fitness value of each swarm particle is computed. For a fitness value greater than the preset threshold value, the fitness value is updated; otherwise, the fitness value is ignored, and the next iteration is continued. The authors have observed that PSO-based approaches have outperformed conventional data-mining-based approaches. The authors Ali et al. [33] have proposed an improved intrusion detection system based on Fast Learning Network and PSO. A fast learning network is a parallel connection of a single hidden layer feedforward network and a three-layer feedforward neural network. A proper weight exploration is indispensable for an appropriate Fast Learning Network (FLN). The results of the work demonstrate an increase in accuracy with an increasing number of neurons in the hidden layer. In the work of Aburomman et al. [34], the authors have addressed the problem of appropriate ensemble construction using PSO. In the literature, it is established by many authors that the ensemble approach can improve the classification results. The authors have proposed an expert system representing a collection of five binary classifiers. Each of these classifiers generates together a binary set of response vectors. Six k-NN and six SVM were trained on the same set of training sets. Three new ensembles were created using the proposed PSO and meta-optimized PSO. The decisions of all twelve experts are considered by the ensembles to reach a final conclusion. Through the experimental results, the authors have established that the classification results can be improved by combining the results of different experts into one by ensemble.

#### 2.2.3. Firefly Algorithm

Firefly Algorithm (FA) is another swarm-intelligence-inspired algorithm from the blinking behavior of fireflies. FA basically uses three rules, which are listed below [35].

- Unisex: One firefly is attracted to another, irrespective of sex.
- The attractiveness and the brightness are proportional. A less bright firefly always moves towards a brighter one.
- The objective function determines the brightness of a firefly.

Different parameters are used to define the attractiveness of a firefly. If β is used to indicate the degree of attractiveness, then it can be defined by

(1) $$\beta =\beta_{0}e^{-\gamma r^{2}}$$

$\beta_{0}$ indicates the attractiveness value at a distance of 0 (*r* = 0). A firefly *i* is attracted to another firefly *j* of higher brightness and moves in the direction of *j*. The direction of motion is given by

(2) $$x_{i}^{t+1}=x_{i}^{t}+\beta_{0}e^{-\gamma r{{}^{2}}_{ij}}\left(x_{j}^{t}-x_{i}^{t}\right)+\alpha_{t}\epsilon_{i}^{t}$$

**Figure 3:**
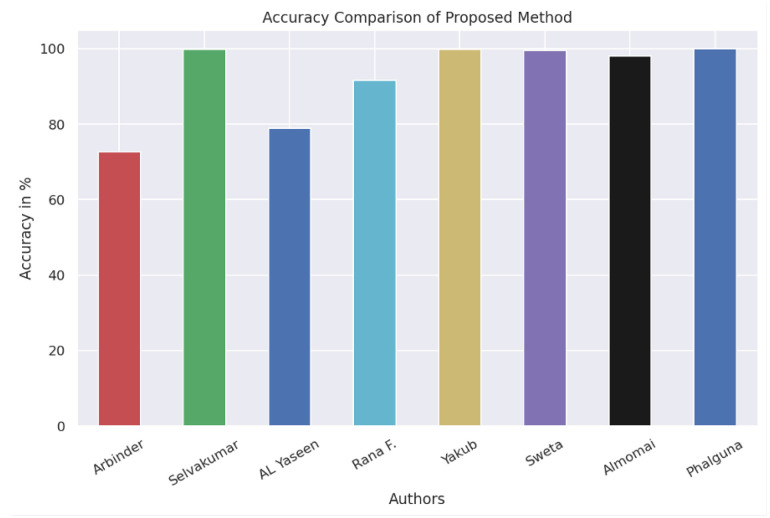
Comparative bar chart for different implemented methods.

$x_{i}^{t+1}$ is the new position of the firefly, i.e., it is the position of the firefly *j*. The second part of the equation is the attractiveness factor, which could be controlled by a factor β. The third term indicates the amount of randomization in the movement of the firefly, with α as the randomization parameter. The ϵ indicates a vector of random numbers generated from a Gaussian distribution. Most of the work of the Firefly Algorithm in IDS is used for feature selection. The authors Selvakumar et al. [36] used FA for FS in building their proposed IDS. Their work represented each firefly as a binary vector and each feature vector consisting of ‘D’ corresponding to the number of fireflies. The firefly with less accuracy moves towards the firefly with high accuracy, and the distance between them is computed. The number of features to adopt or drop is dynamic and is decided by the FA. The KDD’99 dataset is used for training, and the results demonstrate that with all 41 features, the detection accuracy of DoS attack stands at 93%. However, using selected features based on FA and a feature size of 10, the accuracy increases to 99%. In the work of Al-Yaseen et al. [37], the authors have used the Firefly algorithm for feature selection followed by SVM classifier in NSL-KDD dataset. Arivinder et al. [38] have used the Firefly algorithm for the number of k-means cluster formation of the attack groups in NSL-KDD. Rana et al. [39] have used the Firefly algorithm for feature selection and Naive Bayes learning on the NSL-KDD dataset. Saheed et al. [40] have used the Firefly Algorithm for feature selection with Random Forest Classifier on the UNSW-NB15 dataset. Bhattacharya et al. [41] have used the Firefly Algorithm for feature selection and XGBoost classifier on the Kaggle IDS dataset. Almomani et al. [42] have used the Firefly Algorithm for feature selection and SVM together with J48 classifier on the UNSW-NB15 dataset. Shandilya et al. [43] have used the Firefly Algorithm for the selection of nodes that could be monitored in a network. The authors have used SVM classifier on the NSL-KDD dataset. Phalguna et al. [44] have used Random Forest Classifier on the NSL-KDD dataset. The bar chart comparison of the results is shown in Figure 3.

### 2.3. Artificial Immune System Based IDS

Artificial Immune System algorithms are inspired by the human immune system. One of the popular theories proposed is the Negative Selection Algorithm (NSA), where the body attains the ability to distinguish its own cells from foreign cells. Two types of cells are important during the fight against the antigens: T-cells and B-cells. In a negative selection algorithm, the objective is to provide tolerance to self-cells, where immune systems adopt the ability to react to antigens. Any T-cells that react against self-proteins are destroyed by the thymus gland.

This behavior is adopted in designing IDS in the network. Kim et al. [45] presents an Artificial Immune System-based Network intrusion detection system, where there are two IDS in the proposed model—one primary and the other secondary. The primary IDS is responsible for generating various detector sets. The secondary IDS is the local host in the network. The detectors in the secondary IDS are certain background processes that monitor if any non-self-network pattern profile is established. If yes, then those connections are reconsidered. The authors have also used a detector evolutionary process based on the gene evolution process. In the work of Seresht et al. [46], an agent-based approach with inspiration from AIS is proposed for a distributed intrusion detection system. The proposed system MAIS-IDS is a composite system that analyses the traffic of network as well as the host. The analysis of network traffic is done at the virtual machine level. The hypervisor is KVM (Kernel-based Virtual Machine) with three virtual machines running on it. For improvement in the population, clonal selection algorithms are running on each virtual machine. The agents perform the communication with each other in a manner inspired by the agents in the human body. The evaluative results demonstrated that MAIS-IDS reduced the log of false alarms significantly due to the implementation of effective multi-agent communication and collaboration between virtual machines. In the work of Suliman et al. [47], the authors use AIS to detect intrusion on a network. However, they considered two categories of attacks, namely DoS and probing. In the experiment work, each cloned antibody is mutated for an optimal solution. This approach establishes a detection accuracy rate of 99%.

## 3. Plant Defense Mechanism

**Figure 4:**
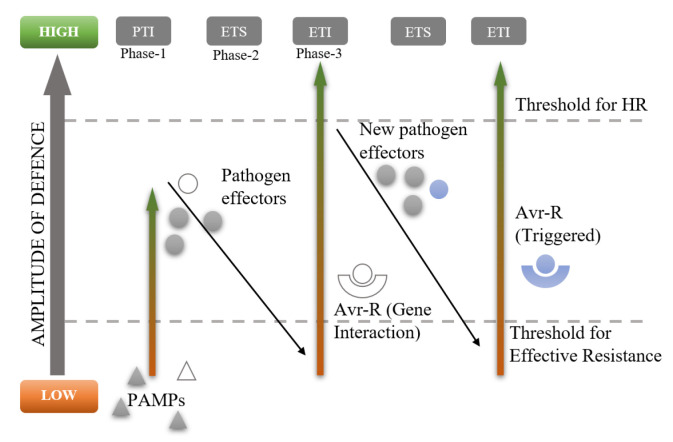
The zig-zag plant defense model [31].

The literature review indicates that plants have a very well-established defence mechanism. The amplitude of defence triggered by plants rises in a zig-zag pattern with the count of pathogen effectors (Figure 4) [31,48]. The authors Sharma et al. [49] proposed and implemented a plant-inspired network attack detection and response model. It’s a three-layered model, similar to how plants defend themselves against pathogens.

Plants have a huge set of receptors that are similar to gene coding that evolved over the years, and they are used to detect any existing pathogens intruding into the plant body. These receptors are referred to as Pattern Recognition Receptors (PRR). Whenever the PRRs detect a pathogen inside a plant’s body, certain preliminary primary defence activities are triggered by plants, such as the closure of stomata to stop the further entry of pathogens or deposition of callose for thickening the cell wall [50].

**Figure 5:**
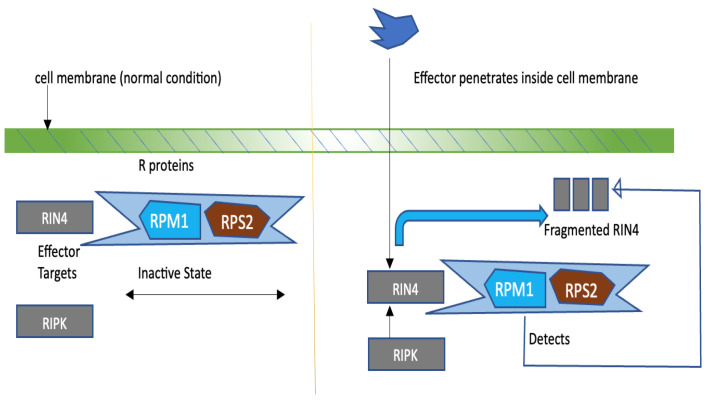
Guard model in plants.

Figure 5 displays a pathogen that has successfully breached the first layer of defence of plants—Pattern Triggered Immunity (PTI), targeting specific proteins in the plant cell. Proteins that guard these targeted proteins are called Guard Proteins.

In normal circumstances, the ‘R (RPM1/RPS2)’ proteins are in an inactive state (Figure 5). If any pathogen successfully breaches the primary layer of defence and cleaves the critical protein such as ‘RPM1-interacting protein 4’ (RIN4), it then results in RPM1-induced protein kinase (RIPK), a mediated phosphorylation of RIN4. Phosphorylated RIN4 is detected by RPM1, resulting in its activation (R protein).

This process also actuates the generation of certain other molecules, such as azelaic acid, for information propagation of a pathogen from the infected area to the distal parts of the plant. High pathogen infection results in the generation of a high amount of ‘R’ proteins. These ‘R’ proteins, later on, trigger the final stage of immunity of killing the local region of the plant such that infection cannot spread from it. This process is also known as the ‘Hyper Response’ (HR) of the plant.

### Induced Systemic Resistance in Plants

The non-circulatory vascular system in plants can carry information from one end to another distal part of the plant [51]. This process is termed SAR (Systemic Acquired Resistance). There have been discovered changes in the metabolic reactions in plants during primary pathogen infection. Consequent to the primary pathogen detection, a number of molecular syntheses take place, such as the generation of Jasmonic Acid (JA), SA (Salicylic Acid), and Azelaic Acid (AZA). They are either synthesized for immune response or transmission of signals to other parts. SA by itself is non-mobile and therefore converted into a mobile signal MeSA (Methyl Salicylic Acid). MeSA flows from one part through the phloem to another part. Accumulation of SA induces secretion of PR (Pathogen-related) proteins with antimicrobial properties, chromatin modifications to generate immune-related genes and genetic recombination for immune memory in plants. It is, therefore, significant to observe that different agents are synthesized and they coordinate among themselves for immune-related activities and signal transfer.

## 4. Salamander Limb Regeneration

Limb regeneration in salamanders goes through different morphological operations [51]. The epithelial cells cover the exposed tissue, forming a wound epidermis. This activity triggers the formation of blastema from the stump tissue. The wound epidermis is formed through the migration of basal epidermal cells.

**Figure 6:**
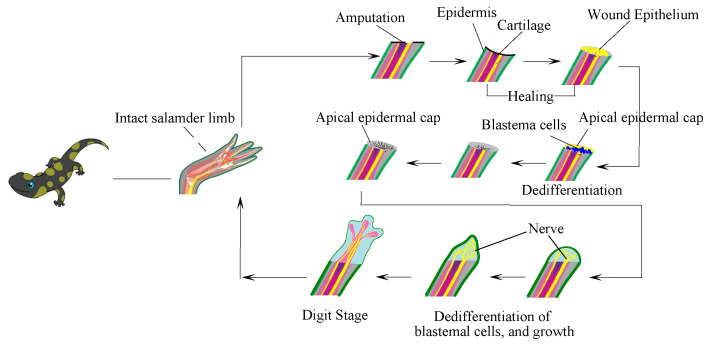
Elements in salamander limb regeneration.

**Figure 7:**
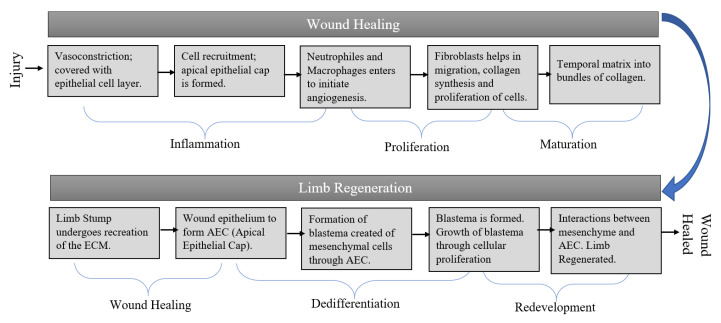
Molecular metabolism for wound recovery and limb regeneration in the salamander.

Along with the accumulation of more blastema cells, thickening of the epidermis is observed, allowing the formation of the apical epidermal cap (AEC) (Figure 6). All the underlying tissues undergo remodeling of the Extra Cellular Matrix (ECM). The moment there is an injury to a body part of a salamander, the ‘vasoconstriction’ process starts, which intends to shrink the nerve deviated to the wounded site and helps in blood clotting. Cell recruitment starts along with the release of growth factors for a quicker process of wound healing [52]. Cells such as neutrophils and macrophages migrate to the wound area. Migrating fibroblast produces growth factors that help in the proliferation of cells. These types of cells in the salamander that migrate to the wound site carry different positional identities. The entire process is presented in Figure 7.

The positional identities help in the formation of the blastema (i.e., the early stage of limb formation). It is not possible to regenerate each body part of the salamander as they are only stored. Once the cells start migrating from different parts of the body, an ECM [52] is formed. In the presence of a deviated nerve to the wound site, the amputated limb starts forming blastemas and finally forms into a fully shaped limb. However, in the absence of any deviated nerve, the blastemas formed regress, and eventually, the scar is healed. This idea of ECM and cell migration is drawn as an inspiration, and a similar analogy is used to propose a data recovery method.

## 5. An Overview of IDS Taxonomy for Detection and Mitigation of Attacks in Network

Machine learning algorithms are increasingly being utilized in IDS to detect and prevent cyber-attacks. The taxonomy of machine learning algorithms used in IDS can be classified into three main categories: supervised learning, unsupervised learning, and reinforcement learning. Supervised learning algorithms utilize labelled data to train a model to identify different types of cyber-attacks. These algorithms require a large amount of labelled data to be effective, and the accuracy of the model depends on the quality and diversity of the training data. Some of the commonly used supervised learning algorithms in IDS include decision trees, random forests, logistic regression, and support vector machines. Unsupervised learning algorithms, on the other hand, do not require labelled data and instead, use clustering or anomaly detection techniques to identify patterns in the data. These algorithms are particularly useful in identifying unknown attacks or zero-day attacks that have not been previously encountered. Some of the commonly used unsupervised learning algorithms in IDS include k-means clustering, hierarchical clustering, and Gaussian mixture models. Reinforcement learning algorithms are used in IDS to dynamically adjust the system’s behavior based on feedback received from the environment. These algorithms work by optimizing a reward function that encourages the system to take actions that maximize the reward. Reinforcement learning algorithms are particularly useful in IDS, where the environment is constantly changing and the system needs to adapt to new threats.

**Figure 8:**
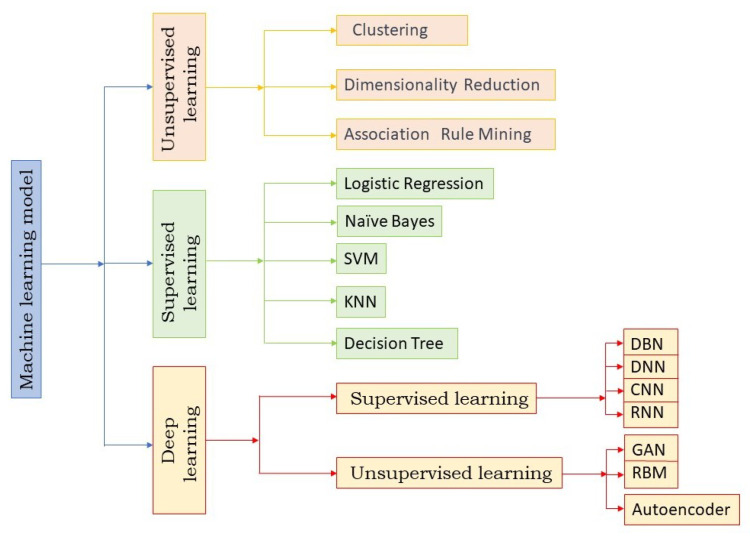
Taxonomy of different Machine Learning algorithms used in IDS.

In addition to the above taxonomy, ensemble learning algorithms are also commonly used in IDS. Ensemble learning combines multiple models to improve the overall accuracy and robustness of the system. This is achieved by aggregating the predictions of multiple models to produce a final prediction. Some of the commonly used ensemble learning algorithms in IDS include bagging, boosting, and stacking. Figure 8 represents the taxonomy of machine learning algorithms used in IDS.

Over the past few decades, a vast array of techniques have been proposed for IDS. In the following discussion, we will focus on some of the cutting-edge methods that utilize unsupervised algorithms. These innovative approaches enable IDS to identify and respond to previously unknown threats in real time, providing unparalleled cybersecurity protection. Verkerken et al. [53] propose an innovative approach to detect malicious behavior on a network using flow-based features and anomaly-based detection. Four unsupervised methods are evaluated, with two of them utilizing a self-supervised learning approach. To evaluate the effectiveness of the proposed models, a modern and realistic dataset, CIC-IDS-2017, which contains a diverse range of attack types, is used. The study assesses the models based on their classification performance and computational complexity, highlighting the potential of this approach in accurately identifying and mitigating security threats in complex and dynamic network environments. Saheed et al. [54] proposed UNIDS, a cutting-edge Unsupervised Network Intrusion Detection System designed to detect unknown network attacks without relying on signatures, labelled traffic, or prior training. UNIDS employs a groundbreaking approach based on Sub-Space Clustering and Multiple Evidence Accumulation techniques to identify various types of network intrusions and attacks, including DoS/DDoS, probing attacks, worm propagation, buffer overflows, and unauthorized access to network resources. The authors tested the system on three distinct traffic datasets, including the widely-used KDD99 dataset, as well as actual traffic traces from two operational networks. The results demonstrate the remarkable ability of UNIDS to pinpoint and respond to previously unknown threats, making it an invaluable tool for ensuring network security in today’s constantly evolving threat landscape.

Ghosh et al. [55] propose an effective Intrusion Detection System (IDS) that utilizes a Logistic Regression (LR)-based classifier, and selects relevant features from the NSL-KDD dataset. To reduce the memory space and learning time required, a feature selection technique is necessary. The study employs the Genetic Algorithm (GA) approach to select a number of feature sets, based on a novel fitness score using Mutual Correlation. The fittest feature set is chosen from among these feature sets using the proposed Best Feature Set Selection (BFSS) method. LR-HIDS [56] is a Host-based Intrusion Detection System (H-IDS) aimed at safeguarding virtual machines in cloud environments. This approach involves selecting critical features of each class via logistic regression and improving their values using the regularization technique. The author uses a combination of three classifiers, including neural network, decision tree, and linear discriminant analysis with the bagging algorithm for classifying various attacks. The model was trained and tested on the NSL-KDD dataset, with a Cloudsim implementation. The results of the simulation demonstrate an acceptable level of accuracy (approximately 97.51%) for detecting attacks against normal states, surpassing other methods tested.

Amine et al. [57] propose a Real-time Dynamic Three-stage Intrusion Detection System (RDTIDS) that integrates multiple classifier approaches that employ decision tree- and rules-based concepts, including REP Tree, JRip algorithm, and Forest PA. The first and second classifiers utilize input features from the dataset to classify network traffic as either Attack or Benign. In contrast, the third classifier uses features from the initial dataset and the outputs of the first and second classifiers as inputs. Through experimental evaluations using the CICIDS2017 and BoT-IoT datasets, the RDTIDS demonstrates superior performance in terms of accuracy, detection rate, false alarm rate, and time overhead when compared to state-of-the-art intrusion detection systems. These results validate the effectiveness of the RDTIDS in enhancing network security and protecting against potential cyber threats. Kruegel et al. [58] present a novel approach to optimize the rules-to-input comparison process in intrusion detection systems using decision trees that leverage machine learning principles. Specifically, the authors demonstrate the effectiveness of their mechanism in improving the processing speed of Snort, a widely used open-source network intrusion detection system. The proposed solution is not limited to Snort, but can also benefit other types of intrusion detection systems such as host-based and network-based ones, as well as packet filters and firewalls.

Li et al. [59] present a novel approach to intrusion detection in wireless sensor networks by utilizing the K-nearest neighbor (KNN) classification algorithm. This approach enables the system to identify anomalous nodes based on their abnormal behaviors and distinguish them from normal nodes. The paper outlines the design and implementation of the proposed intrusion detection system, which effectively enhances the efficiency and speed of intrusion detection by optimizing the Ad hoc On-Demand Distance Vector Routing (AODV) protocol. Wazirali et al. [60] propose a novel semi-supervised technique for improving the performance of Intrusion Detection Systems (IDSs) by reducing the false alarm rate and increasing the detection rate. The approach involves using a k-nearest neighbor (KNN) algorithm with hyperparameter tuning and five-fold cross-validation in semi-supervised learning. Specifically, for each unlabeled data point, the KNN algorithm is used to identify its k-nearest neighbors in the training set. Then, statistical information derived from hyperparameter tunings of these neighbors, such as the number of neighbors belonging to each class, distance metric, and distance weight, is used to classify the new data as either normal or attack class. The NSL-KDD dataset is used to evaluate the proposed approach, and simulation results show that it outperforms existing IDS-based KNN algorithms. Aburomman et al. [34] introduces an innovative approach to constructing ensembles of classifiers for intrusion detection by utilizing weights generated through Particle Swarm Optimization (PSO). To further enhance the performance of PSO, a meta-optimizer in the form of Local Unimodal Sampling (LUS) is employed to optimize the behavioral parameters. The empirical analysis is conducted on five random subsets of the widely used KDD99 dataset, where ensemble classifiers are constructed using the proposed PSO-based method as well as the Weighted Majority Algorithm (WMA) approach. According to the authors, the proposed method yields higher classification accuracy compared to the WMA approach, thereby establishing its superiority in terms of ensemble construction for intrusion detection.

Liao et al. [61] presents a novel CNN-based model for detecting Denial of Service (DoS) attacks in the KDD and CSE-CIC-IDS dataset. The KDD dataset includes four categories of attacks, namely DoS, U2R, R2L, and Probing. In contrast to previous deep learning-based KDD studies, the current study focuses on DoS attacks and performs detection for various types of attacks in the same category. The authors employed an up-to-date IDS dataset that includes advanced DoS attacks such as DoS-Hulk, DoS-SlowHTTPTest, DoS-GoldenEye, DoS-Slowloris, DDoS-LOIC-HTTP, and DDoS-HOIC. Two types of intrusion images, RGB and grayscale, were generated, and a CNN model was designed by considering the number of convolutional layers and kernel size. To evaluate the proposed model’s performance, the authors created 18 different scenarios by varying hyperparameters such as the type of image, the number of convolutional layers, and kernel size. The model’s binary and multiclass classifications were evaluated for each scenario, and optimal scenarios with better performance were suggested.

Abu Al-Haija et al. [62] develop a new intelligent and autonomous deep-learning-based detection and classification system for cyber-attacks in IoT communication networks, called the IoT-based Intrusion Detection and Classification System using Convolutional Neural Network (IoT-IDCS-CNN). The IoT-IDCS-CNN system is composed of three subsystems: a feature engineering subsystem, a feature learning subsystem, and a traffic classification subsystem. The feature engineering subsystem is responsible for extracting relevant features from the raw data, while the feature learning subsystem uses these features to train the neural network. The traffic classification subsystem is then used to classify network traffic as normal or anomalous. The authors evaluated the system using the Network Security Laboratory-Knowledge Discovery Databases (NSL-KDD) dataset, which includes all the key attacks in IoT computing.

Roy et al. [63] implement a Long Short-Term Memory Recurrent Neural Network (LSTM RNN) to analyze Internet of Things (IoT) traffic patterns for the purpose of detecting and distinguishing between normal and malicious network behavior. The authors trained the LSTM RNN model using the UNSW-NB15 dataset and evaluated its performance based on various metrics such as recall, precision, False Alarm Rate (FAR), and F-1 score. Their findings revealed that the BLSTM RNN-based Intrusion Detection System (IDS) was highly efficient and accurate in detecting anomalous traffic behavior. However, the authors recommended conducting further experiments on larger datasets of IoT traffic to validate their results.

## 6. Earlier Work and Motivation Forward

**Figure 9:**
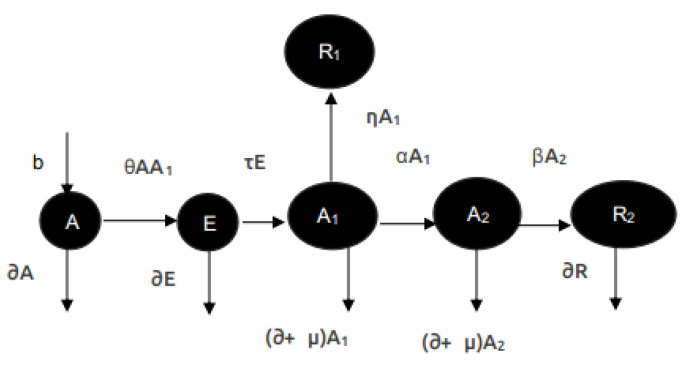
Transmission of malicious program flow in a network.

**Figure 10:**
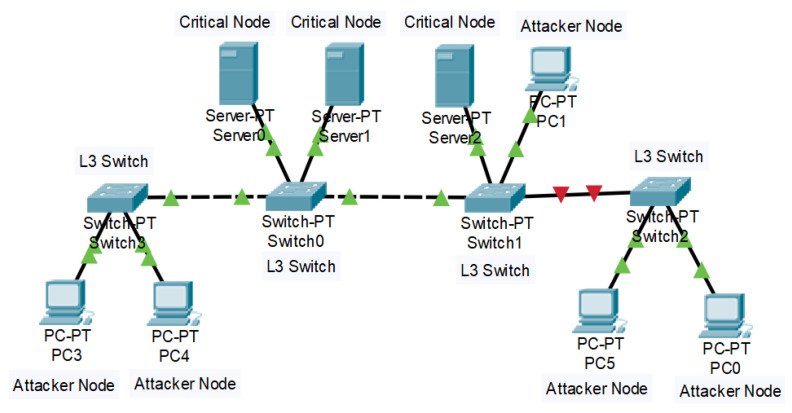
LAN topology.

The first time proposal of the model PIRIDS was done in the earlier work of Sharma et al. [49]. The intrusion detection capacity of PIRIDS was validated against the real-time ssh worm developed to spread over a network. A replication factor of 2 was considered in this experiment. The justification for consideration of replication factor two can be found out from Figure 9. ‘*A*’ is the number of susceptible systems exposed to threat in a network. At a given instant of time, the number of systems newly included is ‘*b*’, and ‘*d*’ is the natural crush of systems on the network. For ‘*N*’ systems in the network for which ‘*n*’ systems (*n* < *N*) are under attack are placed in $A_{1}$. For an infectious rate θ, the infection will spread from class $A_{1}$ as well as *A*. The change in the number of systems in *A* is therefore given by

(3) $$\frac{dA}{dt}=b-\left(\theta AA_{1}+dA\right)$$

if η is the removal rate of systems due to hypersensitive response or threat by an attacking agent then $R_{1}$ is the set of the removed class. For ‘*k*’ the number of systems that are the probability of systems not to be recovered then n−k systems can still infect other systems. This set of remaining nodes is the infectious class $A_{2}$. It is therefore important that the recovery mechanism is triggered before the infection spreads to at least two nodes in the network. This is because if both nodes retaining a copy of the critical resource fail, then recovery of the file would be impossible later in the network. A clear picture of the test bed implementation of the attack scenario is also presented in Figure 10.

**Figure 11:**
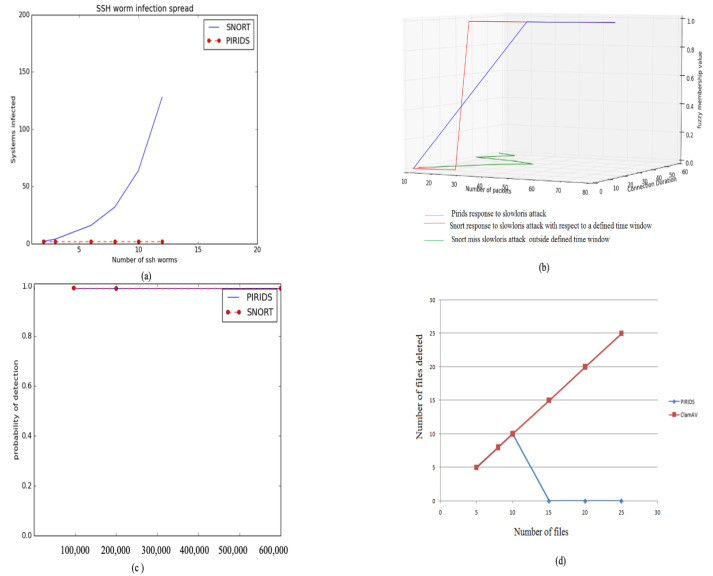
Experimental results [49]: (**a**) SSH worm infection spread; (**b**) Slowloris detection; (**c**) TCP-Syn flood attack; (**d**) File deletion comparison under Ransomware like attack.

In the work of Fioriti et al. [64], a graph network representing the scale in which an SSH worm can spread is represented. Figure 11a shows the early detection of SSH worm infection spread in comparison to open-source IDS Snort. The validation of PIRIDS against Slowloris attack was also explored and the comparative results in comparison to Snort are shown in Figure 11b. Due to this early response by PIRIDS, the critical resource alteration in a system by Ransomware or similar attacks was seen to be significantly low. This is indicative from Figure 11. Figure 11 summarizes earlier results of PIRIDS [49]. However, the earlier work did not explore the possibilities of a nature-inspired network recovery model subsequent to an attack that could completely evade human intervention for the network to revive. This further motivated exploring different recovery agents in nature. One such creature is the salamander. With its amazing morphological operations of regeneration, it proved to be a substantial subject of the present study. The limb regeneration of the salamander was taken as an inspiration to propose a network node recovery model. The proposed model and the experimental findings are presented in this work. The results of the earlier PIRIDS model are also augmented with the complexity analysis in terms of time and space for attack detection and data recovery in this paper.

## 7. Proposed Model of Data Recovery Inspired from Salamander

The proposed method is a distributed participatory data storing and recovery system. The resources useful for the user are marked ‘critical’. The content of a CD (Critical Directory) can be user-defined files, system files, services files, etc. However, the focus of the work is restricted to user files. The working functionality of the proposed method is an inspiration drawn from the limb regeneration in salamanders [65]. Certain features from this organism are adapted to obtain a model, as shown in Figure 7. Some of the agent programs considered are discussed below.

### 7.1. Monitoring Agent

In our proposed system, we consider the user’s files as a critical resource. Ransomware [66] targets user files such as PDFs or Microsoft Word documents. To detect any malicious operation on the critical resources, we must be aware of the changes happening to the files and find out if the changes are malicious or legitimate. In the implementation, the ‘fanotify’ and ‘inotify’ functionalities of the Linux kernel are used to monitor the read, write and delete operations of the files. These functionalities of the Linux kernel provide library bindings to monitor file system activities. The method “PIRIDS” discussed earlier, notifies this monitor agent about the suspected process. The monitor agent checks the entropy of all those files. If the entropy is high, the files are encrypted [67] and then another agent, “Resource request agent” is activated for requesting recovery of the resource from the peers.

### 7.2. Backup Agent

The backup agent sends backups of critical files to the network peers. Once the network peers to whom backup copies are to be sent are identified, an extracellular-like matrix (ECM) of salamander is generated in which each row entry comprises two columns. The first column is the concatenated entry of the IP address and MAC address of the peer. This is done because, in a network with dynamic IP address assignment, the IP address may vary; however, the MAC address entry remains constant for a given system in the network. The second column is the relative or absolute path entry of the critical resource that is to be distributed. During the course of implementation, a replication factor of two is considered, i.e., each backup copy is transmitted to two peers in the network.

### 7.3. Multicast Agent

The multicast agent is responsible for multi-casting its identity to its peers. The identity of a peer is necessary to all other peers and vice versa to participate in the backup and recovery packets. The multicast of this information is accomplished in the form of a “HELLO” packet. The critical peers in a network are part of a common multicast group. They exchange their identity using multicast “HELLO” messages. The identity of a node is its own MAC address, which is 48-bit in size. The “HELLO” message carries the source MAC and IP addresses as the data stream, which is extracted by the peers and stored in a “nodes.db” database file. The backup agent in the receiving peer creates a directory by the received MAC value. This directory is used to store all the files received from that specific node. Once the “nodes.db” is formed, every CN transmits each critical file from its own CD with a replication factor of 2.

Whenever this is detected by “PIRIDS”, it notifies the monitor agent that computes the entropy of the file. Shannon’s entropy [68] on a normal file is less than a file with high randomness. An encrypted file always has high randomness and therefore, higher entropy. If the calculated entropy is high, the monitor agent notifies the file recovery process to its peers. The peers start acknowledging the files along with their path structure from the MAC directory. Once all the files are received, the requesting node forms a file matrix (FM). The victim node then executes the command to create the directory structure and copies all the files there, thus restoring the original CD structure. Agents are programs created to meet certain objective tasks. Thus, activating an agent means bringing the agent program to life, i.e., the process in execution. The corresponding Algorithm 1 is a representation of the data recovery method. Where IP addresses in DHCP network may change, the MAC address acts as the identifier. Since the MAC address of a system is always unique, this value is transmitted as application data. The need to communicate the MAC address as application data is because MAC broadcast is never forwarded by routers in a network.
**Algorithm 1:** Proposed Method for Resources Recovery after Ransomware Attack
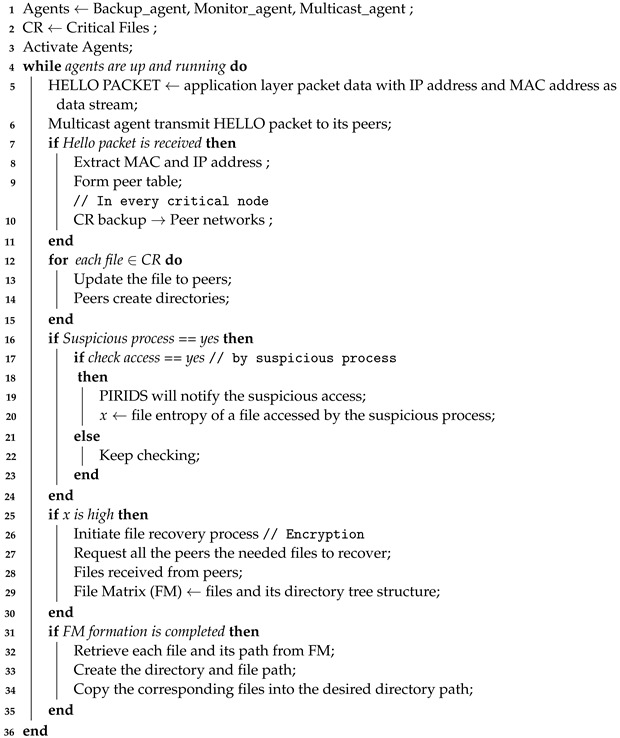


## 8. Understanding Data Recovery to a Ransomware-like Attack

To understand the data recovery process, it is assumed that the ‘Server Message Block’ (SMB) protocol [69] is active in the network, and there are no previously known receptor agents running on the system. The recovery would be discussed in the circumstances of ‘WannaCry’ [70]-like ransomware. Once the WannaCry ransomware resides on a victim system, the worm tries to spread to other systems through SMB discovery. The malicious program creates two threads out of which the first thread scans the host on LAN and the second tries to connect to port 445 on the LAN. Once installed, the installer will extract an embedded file into the same folder necessary for further execution.

Once the WannaCry program establishes a backdoor and is connected to the C2 servers [71], it starts scanning the entire drive and also network drives for files with specific extensions for encryption. As the file encryption starts, the fuzzy vector for this process exceeds the threshold value and the process is terminated. The entropy of the files is computed, as seen in step 16 of Algorithm 1 in [49]. If the entropy value is high, then the file recovery (Multicast request) is initiated from the compromised CN (step 19). Once this request reaches the peers, the backup files in the corresponding receiving nodes are sent back to the sender. The response carries the file name together with the directory path. On receiving a response to different files from different CNs in the network (step 21), a file recovery matrix-like ECM is formed (step 22). Once the formation of ECM is complete, the directory tree structure is created and the file is copied back to its original position.

## 9. Attack Detection Complexity of the Proposed Model

The performance complexity of the proposed PIRIDS model is proportional to the number of detector agents in layer 1 and layer 2 of the model. If *k* number of detectors are present in level 1, then different numbers of attacks that could be detected at level 1 can be given by

(4) N=k∗L

where *N* is the number of attacks detected, *k* is the integer value factor that indicates how many different attacks each agent is programmed to detect and *L* is the number of detector agents. The level 2 defense layer has a process monitoring agent that detects malicious processes that have successfully breached level 1 defense of PIRIDS. The fuzzy value of this process changes with time with its increasing malicious activity (sigmoidal behavior) and whenever the threshold value is exceeded, the process is terminated.

It can, therefore, be stated as

(5) $$y_{i}=f\left(x\right)$$

where $y_{i}$ is the fuzzy computation of a process at a given time,

(6) $$Y=\Sigma y_{i}$$

where, f(x)=0 for x<=a, a is a lower limit value until fuzzy update due to file operation by the process;

1. $f\left(x\right)=2{\left[\left(x-a\right)/\left(c-a\right)\right]}^{2},c$ is the threshold to consider a process malicious;
2. f(x)=1−2[(x−c)/(c−a)]2,b<=x<=c,b is the point where the membership degree tends to rise significantly;
3. f(x)=1,x>=c.

If *Y* is greater than the threshold, then the process will be considered malicious.

## 10. Experimental Results and Analysis

The experiment is carried out in a LAN framework as shown in Figure 10. All the servers hosting certain services or repository of resources are the CNs in the implementation. The experiment is carried out in a LAN-Testbed with the following basic configurations:

- Linux systems with Ubuntu 16 OS.
- CPU Intel i5 processor.
- DLink Layer2 switch for the LAN network.
- 12 GB RAM.
- Python 2 and 3 for the program agents.

**Figure 12:**
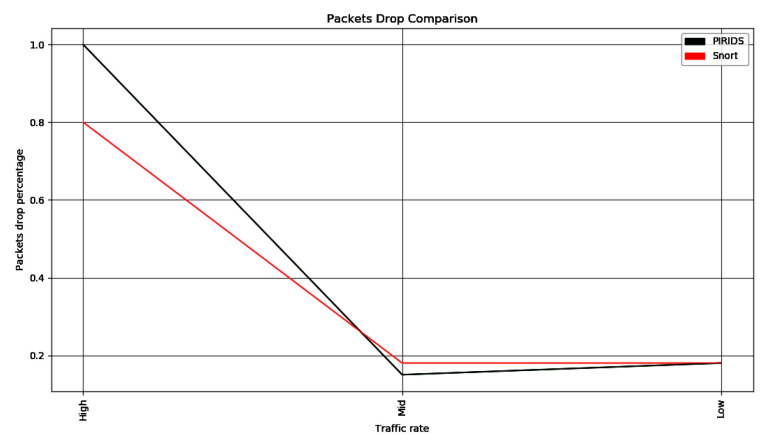
Packet drop comparison between Snort and PIRIDS.

The packet drop ratio comparison between Snort and PIRIDS is shown in Figure 12. With high network data traffic, the packet drop ratio performance of Snort is better than PIRIDS. This is because of the header data rule filtering of Snort in comparison to the header and application data filtering in PIRIDS. The above performance measure is in a standard system as mentioned. With higher hardware capacity, the packet drop ratio observation could be better.

A comparison chart with the response time of worm detection in a network and accuracy detection of different attacks is shown in Table 2. It can be observed that the proposed method is significant for the detection of an ssh worm bot in a network with infection spread to less than five hosts in a network. The proposed method also outperforms other peers in terms of Slowloris attack detection in a network and is close to the work of Abushwereb et al. [72]. However, the work of Abushwereb demands computational resources in the training stage and later in the preprocessing of the real-time traffic. This demonstrates the usability of the proposed model in limited resource constraints and for the detection of resource-specific targeted attacks.

## 11. Data Recovery by the Proposed Model after Ransomware-like Attack

In this section, the data recovery method, as discussed in Section 5 and Section 6, is discussed from an implementation point of view. It is observed in Algorithm 1 that the fundamental aspect of the data recovery is communication between the multicast group of CNs in the network. These communications are nothing but query and response messages that are exchanged between the nodes. The overall communications among the nodes are managed by the multicast agent, backup agent and monitor agent. We use UDP multicast sockets to communicate among the nodes. All the nodes subscribe to the multicast address. This works efficiently in a local area network. After the UDP socket is created, the nodes advertise themselves by sending the “HELLO” packets to the multicast group. All the other nodes of the group receive this information and they create a local directory with the MAC identifier to store the nodes’ critical files. Messages to the multicast group are sent inside UDP packets as a string. The different types of messages used are as follows:

1. HELLO message—used to multicast the node ID, this does not contain a recipient node ID.
2. STORE message—used to notify a peer to be ready to receive backup files to store.
3. GIVE message—used to fetch the critical resources backed up by the peers in the network.

**Figure 13:**
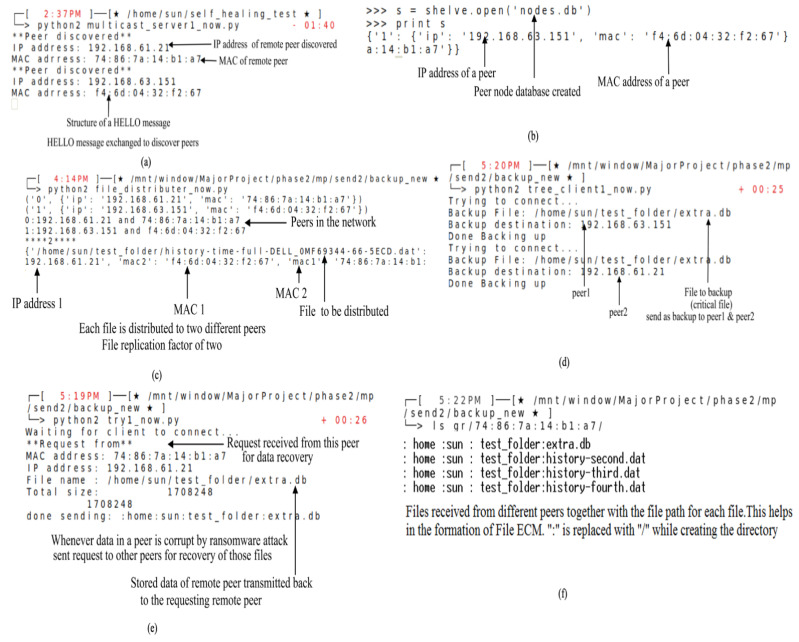
File recovery: (**a**) Multicast Hello Packet; (**b**) Database of critical node peers; (**c**) File distribution to peers for backup-script; (**d**) File distribution to peers for backup-database; (**e**) Backup request from peers and recovery initialization; (**f**) ECM formation after fragments of files received from peers. Note: The ‘star’ symbol in all the figures is the Linux terminal prompt symbol and * is for comments.

The multicast hello packets received by the CNs are shown in Figure 13. The IP address and the MAC address of the system are received as identifiers of the CN. These are stored for future reference. The consideration of the MAC address along with the IP address is important as the dynamically allocated IP of a CN may change any time.

As shown in Figure 13a, the IP address received is ‘192.168.61.21’ with the corresponding MAC as “74:86:7a:14:b1:a7”. The multicast client agent will multicast its IP and MAC addresses, to all other CNs part of the multicast group.

Figure 13b shows the node table database of the peers in the network after their discovery using the HELLO packet. The peer table is “nodes.db”. Once the table with identifier values is created, any CN is now ready for distributing the files from the directory marked as critical. The MAC and IP address concatenated is created as one table entry.

The copy of each file is distributed to two different other CNs, i.e., a replication factor of 2 is adopted throughout the implementation. Figure 13c illustrates that the backup script file ‘distributor.py’ is initiated. File distribution is created by assigning one file to two different peers.

Once the distribution table is ready after the HELLO packet exchange, the data are transmitted for backup. As shown in Figure 13d, the file extra.db is sent as a backup copy to peer 1 with IP address 192.168.63.151 and the second peer with IP address 192.168.61.21. The file extra.db is a critical file. Whenever a node fails, it multicasts to its peers a request to recover the critical file directory.

All other peers who have the corresponding node’s resources start transmitting them. As seen in Figure 13e, the recovery request is received from a peer with an IP address 192.168.61.21. The MAC identifier is carried as a data stream in the request. The file “extra.db” is the stored file of the requesting peer; which is transmitted along with the file path structure, i.e., “/home/sun/test_folder/extra.db” to the requesting node.

The requesting CN receives all the files from other peers. Once it receives the files, it activates a process similar to an ECM in Salamander as discussed in Section 7. Together with the files, the respective path is also received. After that, the requesting node creates a directory tree similar to the one in the original form. As seen in Figure 13f, all the files are received from different peers. The ‘:’ is replaced with a ‘/’ to recreate the file path while generating the CD.

## 12. Performance Comparison of the Recovery Model

**Figure 14:**
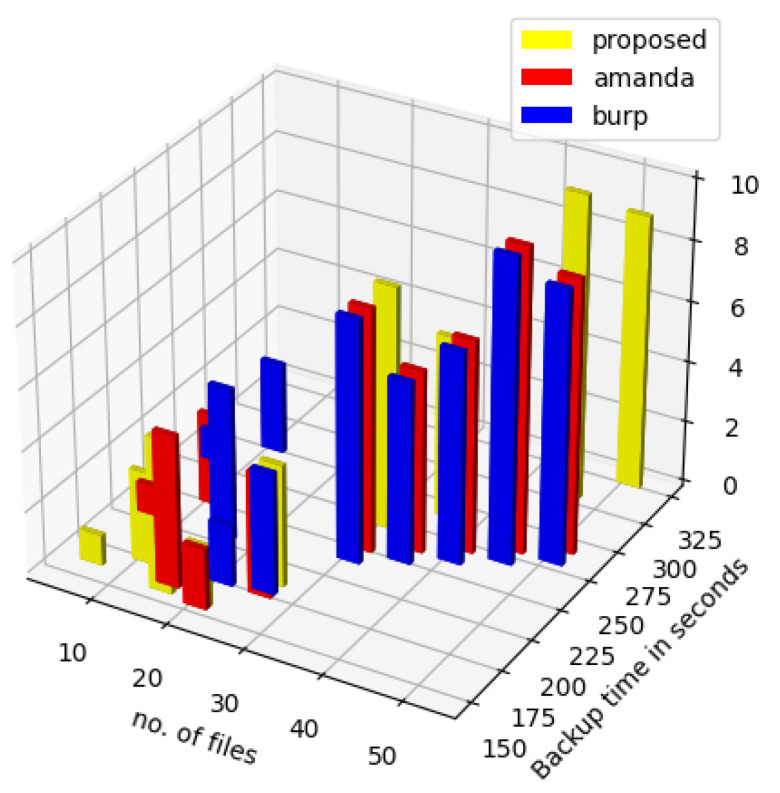
Backup time comparison.

Certain parameters of the proposed method were compared with the respective parameters of Amanda and Burp, which are open-source network backup and restore tools. As seen in Figure 14, the proposed method performs better with respect to data backup time for a lesser number of files. The z-axis indicates the number of backup agents active in the LAN network. The increasing backup time for Burp might be because of the two-phase operations involved in the backup operation. In the first phase, the client scans its file system and sends the metadata information to the server. Metadata includes the path, filename, and system entry type (regular file, directory, etc.). In the second phase, the server reads through the manifest of the previous backup and the present manifest. If the file is not found in the previous manifest, it asks the client to transmit the whole file. If it finds the file, where the modification time is different, it generates a signature of the previous backup and transmits it to the client. The client uses this signature to transfer only blocks that have changed. The backup time in the proposed method is initially flat as indicative of the blue line graph. This is because the metadata exchange time for the file backup is less with a small number of files. However as the number of files increases, the size of the metadata table together with the number of chunks of data for backup increases, thereby shooting up the backup time. In the case of Amanda and Burp, the initial backup time is high because of the checksum calculation the software runs on the initial chunks of data.

### Experiment Setup for Amanda and Burp

The Amanda backup server was run on Red Hat Linux Enterprise Server, and Amanda client software was installed in Ubuntu client systems. The version of Amanda installed is 2.5. Once the software was installed, necessary changes were made to the configurations file for execution, tape management and specifically for non-encrypted backup to save backup time. The same was the case for the Burp backup server. The server of both Amanda and Burp was hosted on a local LAN on a Class C address. Customized changes were made to burp.conf, burp-server.conf files for backup and restore configurations on the LAN.

**Figure 15:**
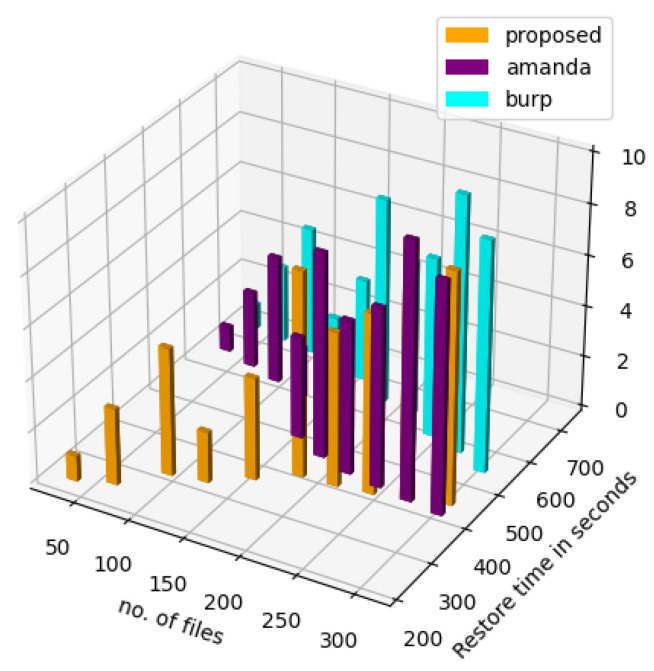
Restore time comparison.

The restore time, as seen in Figure 15 for the proposed method, is significantly better than both Amanda and Burp for a significantly large number of files. The z-axis indicates the number of backup and recovery agents in the network. The delay in Amanda might be because once the restore files are selected, Amanda needs to find the required tape and then look for the backup image, decompress if necessary and then transmit the image over the network to the client. In Amanda tape format, the first tape file is a volume label with the tape volume serial number and date it was written. Each file after that contains one image using 32 KB blocks.

## 13. Time Complexity Analysis for PIRIDS Model

To compute the complexity of the proposed model in Algorithm 1 of this paper, it is translated into the following steps:

1. Initialize all the receptor agents (SQL injection detector, Slowloris detector agent, syn flood detector agent, honeypot agent, ssh worm detector agent, Monitor agent).
2. While all the agents are up and running, do:
  - Receptor agents sniff incoming packets.
  - The feature vector set of the sniffed packet is extracted.
  - The fuzzy membership value of the extracted feature set is computed.
  - The newly computed fuzzy value of the feature vector is added to its previous value.
  - If the new fuzzy value exceeds a defined threshold, then action against the IP address corresponding to the feature vector set is taken and the fuzzy value of the feature vector is distributed to peers.
  - If the threshold is not exceeded, then the fuzzy value of the feature vector is distributed to its peers so that their respective tables could be updated.
  - Monitor agents monitor for the set of processes breaching receptor level detection and interacting with the CD.
  - Fuzzy member of the process interacting with CD is computed and distributed to peers.
  - The entropy of the files in CD is computed and if found a high file recovery process is initiated and the feature vector of the process is distributed to peers.
  - If the number of files with high entropy is discovered, then the system is temporarily terminated from the network to stop further infection propagation.

The resultant time complexity of the PIRIDS model is the sum of the total complexity, as explained below. It is important to note that O(m)+O(n)=O(m+n).

Let us address the independent complexity of each of the receptor agents. Once the complexity of receptor agents is computed, then it could be added to the complexity of the entire sub-steps to compute the resultant complexity.

The time complexity of the receptor agents is shown as follows under 4 sub-sections:

- SQL injection detector: Here, every packet sniffed by the scapy-based sniffing agent is pushed to the parsing function as a parameter. Therefore, it is important to note that the processing is happening for every packet that goes inside the function independently. Inside the program, the HTTP header is extracted and split by the ‘referer’ tag. Once split and stored in a tuple, the tuple entries are searched for a host tag, and the corresponding host entry is parsed for SQL injection tags. The HTTP tag entry is also retrieved for verifying possible redirection to a malicious site. Once the full requested URL is retrieved, then it is searched for possible SQL injection patterns in the URL tag from the SQL injection set of patterns. The program, therefore, uses two iterative loops, one for iterating through the SQL injection set pattern and the other for iterating through the full URL of length ‘*n*’. The size of the URL pattern set is fixed as ‘*k*’. The time complexity is, therefore, O(Kn) or kO(n)=O(n), where k is constant.
- Slowloris detector Agent: The Slowloris detector agent is responsible of detecting an incomplete HTTP header (i.e., non-terminating of HTTP header by \r \n \r \n). Here, the parsing of HTTP header data is done for every packet, and the number of fields in an HTTP header always remains fixed irrespective of the size of the HTTP packet. The Slowloris detector program has a time complexity of order O(K.1)=O(K)=O(1), where ‘*K*’ is dependent on the number of a fixed amount of time instruction and is constant with respect to the input size.
- SYN flood detector Agent: The SYN flood detector agent is responsible for detecting the number of SYN requests arriving at the host in a given time window. The detector agent is programmed for every packet that has arrived to look for the TCP header and if present, it looks for the presence of SYN flag. This takes O(K), where *K* is a constant. For ‘*n*’ number of packets arriving at the node at a given time window, the time complexity for processing TCP/UDP SYN flood is O(n).
- Monitor Agent: The monitor agent is responsible for monitoring any malicious process breaching layer 1 defence and interacting with resources in the CD. Once identified the respective malicious program is terminated and if required file recovery is initiated. The input passed to the monitor agent is the file list of all the resources considered as critical. The fanotify of Linux is used to monitor any update on a file inside the CD. The monitor agent walks through the directories and sub-directories and then finally into the critical files of the directories. Therefore, this could have ‘*n*’ directories and ‘*n*’ files under each directory. The time complexity is therefore: $O(n^{2})$.

The resultant complexity of the PIRIDS model in level 1 (PRR) is the sum of the total complexity of the detector agents. From the complexity of the monitor agent, it can therefore be arrived at

(7) $$O\left(n\right)+O\left(1\right)+O\left(n^{2}\right)=O\left(n+n^{2}+1\right)\to O\left(n^{2}\right)$$

**Figure 16:**
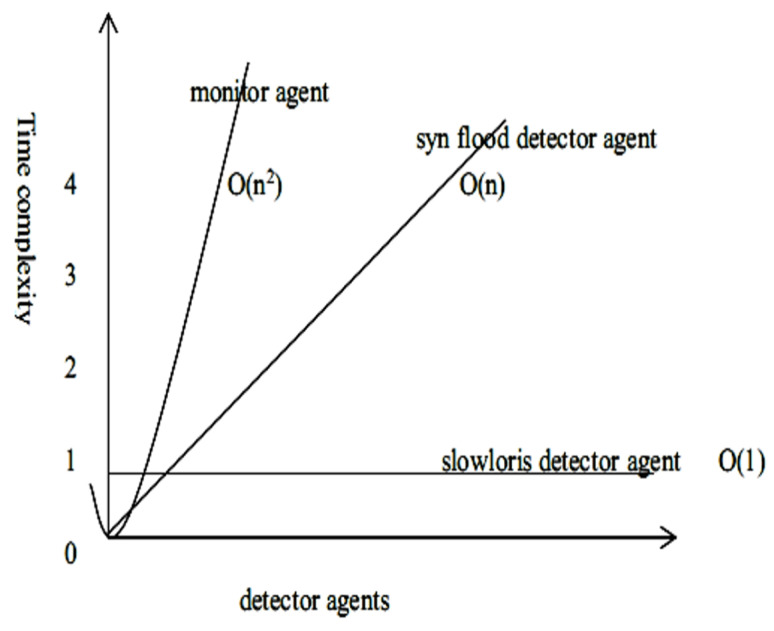
Different time complexity order for different detector agents.

As can be observed from the time complexity analysis above, the order of complexity is constant say ‘*k*’ and therefore O(k), i.e., O(1). It can thus be concluded that the complexity of the PIRIDS detection model is primarily determined by the highest order complexity of the detector agents running in level 1 of the model. Figure 16 shows a different time complexity for detector agents.

Time complexity of the data recovery model: The data recovery model is discussed in Algorithm 1 of Section 7. The recovery agent requests file recovery from its peers, and once the files are received from its peers, the recovery agent builds the FM. Once the FM is built, then the recovery agents create the file as well with the file structure in the FM. There may be ‘*n*’ number of files and, therefore ‘*n*’ entries in the FM. The time order of the complexity of the recovery model is O(n).

## 14. Space Complexity Analysis for PIRIDS Model

**Figure 17:**
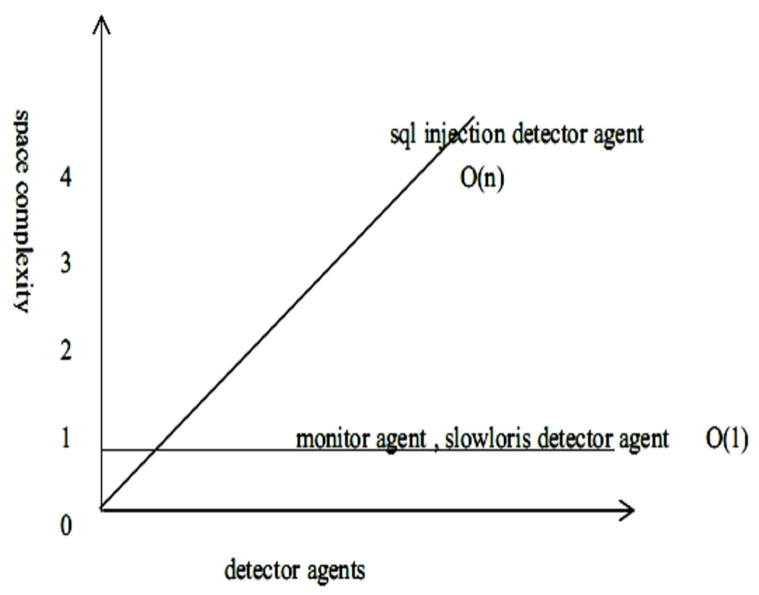
Different space complexity order for different detector agents.

The space complexity analysis computes the memory consumption by the algorithm during run-time. The Slowloris agent extracts the HTTP header from the sniffed incoming packet. The extracted header is stored in a list for parsing. The maximum size of an HTTP header is, however, fixed and therefore, irrespective of the size of the incoming packet, the header size is going to be limited. If the size of the packet is ‘*n*’ bytes, then the list of instruction initialized is of size O(k) where *k* is constant. The instruction initialization in the Slowloris agent takes O(1). Therefore, the net space complexity for the Slowloris agent detector is O(k)=O(1). In the SQL detector agent, from the HTTP header, the URL is extracted for parsing. The extracted URL is stored in a string array equal to the size of the length of the URL. If the length of the URL is ‘*n*’ bytes, then the space complexity is O(n). Since all other instruction takes O(1) space complexity, the net space complexity is O(n). The monitor agent looks for all those processes interacting with the critical resources and for any process interacting with the critical resources, the corresponding fuzzy value is computed and distributed to its peers. In all these processes, the instruction takes O(k) space, where ‘*k*’ is constant, and therefore the space complexity is O(1). Figure 17 shows different space complexity for different agents.

## 15. Conclusions

This work explores how the pathogen detectors in plants evolved over the years and could be taken as an inspiration to design a robust IDS in a network. The naturally evolved a three-layer defence model in plants apart from successfully defending itself against pathogen attacks and also adopts extreme action against itself to stop further causality caused by pathogens. This by itself is very encouraging for the design of a robust response system so that the spread of infection is terminated. The experimental results demonstrate that the proposed model is competitive against other methods found in the literature for the detection of both known and unknown attacks.

The limb regeneration model of salamanders gave insight into an altogether new idea of positional matrices. This idea was carried forward to retain a similar data structure of recovery nodes in networks so that, in case of extreme failure, the data and services could be effectively recovered. The experimental results of the proposed model have demonstrated that both detection and recovery models are effective under different circumstances of network attacks. The packet drop found in comparison to the Snort IDS could be improved by using better hardware. The data recovery method performs well provided there are enough uncompromising critical peers in the network to retain the copies possible for data recovery. Future work, therefore, could be concentrated on improving these aspects of the proposed model.

## Figures and Tables

**Table 1:** Comparison between ML and Evolutionary Algorithms.

Criteria	ML and DL	Swarm and Evolutionary Algorithms
Working principle	Building a mapping between input and output	Finding an optimal solution from a set of population
Output	Trained Model	Optimal candidate solution
The goal of generalization	Aims at minimizing the computational cost of unseen data samples	Aims at minimizing the computational cost of known data samples
Applicability	Primarily used for classification, clustering, regression and feature extraction	Feature set and result optimization

**Table 2:** Comparison of attacks detection.

Authors	Nature of Attack Detection	Response Time/Mechanism	Infection Spread/Accuracy of Detection
Abushwereb et al. [72]	Slowloris attack detection	Multiclass classification	99.9%
Gu et al. [73]	Worm Spread	15 min (average)	>1500 hosts
Singh et al. [74]	Worm Scan	30 min	∼1200 worm detection
Sharma et al. [49]	Worm Scan	5 min (average)	<10 host
Valizadeh et al. [75]	Worm Scan	15 min	<100 host
Singh et al. [76]	Slowloris attack detection	ML approach	91.40%
Sharma et al. [49]	Slowloris attack detection	Time window based signature verification	97%
Singh et al. [76]	Slowloris attack detection	Random Forest	92%
Xu et al. [77]	Slowloris attack detection	Hybrid deep neural network	93%

## Data Availability

Not applicable.
